# Obesity from the perspective of the liver

**DOI:** 10.3389/pore.2026.1612376

**Published:** 2026-07-07

**Authors:** Miklós Csala, József Mandl

**Affiliations:** Department of Molecular Biology, Semmelweis University, Budapest, Hungary

**Keywords:** endoplasmic reticulum, glucose, glycogen, lipotoxicity, NADP

## Abstract

Glucose is at the center of liver metabolism, and together with NADPH, it is an essential factor in hepatic regulation and adaptation to different metabolic states. The liver plays a fundamental role in the development of obesity. Excess food intake, particularly excess glucose, primarily burdens liver metabolism. The hepatocyte-specific glycogenoreticular system, a functional unit based on the tight interaction between glycogen particles and the ER, is central to the storage and distribution of glucose throughout the body. Additionally, the liver converts excess glucose into ketogenic molecules. The liver is the most important site for glucogenic to ketogenic conversion in the body. However, the escape route offered by unrestricted fatty acid synthesis and deposition takes its toll in the long run. An NADPH pool is also a pivotal connection among intermediary metabolism, redox homeostasis, and drug metabolism in hepatocytes. Preservation and recovery of excess glucose is a commitment, which presents a potential pitfall. Overfeeding can lead to pathological consequences by disrupting regulatory mechanisms. The fulfillment of metabolic goals by the liver plays a significant role in the development of various pathological conditions, such as fatty liver, obesity, insulin resistance, and metabolic syndrome.

## Introduction

The epidemic of obesity-related diseases is one of the most important medical, public health and sociological issues of our time, raising serious questions at the societal level, too [1]. Obesity is a complex disorder of nutrient storage. Nutrients can be stored and mobilized in glucogenic or ketogenic form. The liver is the central organ of the body’s metabolism, both in terms of nutrient storage and mobilization, and is also the main site of glucogenic-ketogenic conversion in the body. Obesity can be looked at from several perspectives. Some approach it from an evolutionary point of view, others from the perspective of the gut-liver-brain axis, or even microbial dysbiosis. In this review article, obesity is examined from the molecular aspects of liver function with emphasis specifically on the glucose-NADPH-endoplasmic reticulum (ER)-lipogenesis axis.

The supply and demand of glucose determines the metabolism of the liver. Hepatocytes are able to store glucose in the form of glycogen for the entire body. Ensuring that glucose is available to all cells is the primary task of the liver’s metabolism. With the exception of lipids, substances that are absorbed from the intestinal tract are collected in the portal circulation and carried directly to the liver. Glucose is of unique importance among these compounds [2]. It is the only nutrient that can be consumed by any cell in the body. On the one hand, glucose must be used sparingly because the capacity of its storage is limited and it needs specific precursors to be produced, which are scarce in starvation. As an important part of glucose conservation, the kidneys actively retain it preventing its excretion in urine [3]. On the other hand, excessive glucose exposition is harmful to cells and such glucotoxicity plays an important role in obesity-related diseases [4]. Blood glucose levels are influenced by a number of hormones, of which insulin from the endocrine pancreas is of particular importance [5]. The liver plays a dominant role in lowering as well as raising and maintaining blood glucose levels, both by storing and processing excess sugar and by replenishing it from storage or synthesis in the event of a shortage, thus flexibly adapting to changes in the metabolic needs of the body. It is therefore crucial to maintain the carbohydrate reserve and carbohydrate-synthesizing capacity of the liver.

Besides the unique importance of glucose metabolism, another essential aspect of liver metabolism is the NADPH pool, i.e., the common source of reducing power in the cells. The fundamental hepatic functions require reducing power [6], so in addition to being a reliable glucose supplier, the liver must also maintain the supply of electrons, i.e., hydrogen atoms, in a largely changing availability of the sources. While the capacity of storing glucose in the form of glycogen is largely limited, a virtually unlimited amount of the reducing power can be efficiently stored in the form of fat. Therefore, in the absorptive, fed state, when more glucose is available than the body can store, the liver freely collects the hydrogens of glucose and synthesizes fatty acids to save as much as possible. However, in starvation, glucose is precious and decreasingly available, so the reducing power of stored fatty acids is being utilized.

The common features of liver functions are synthesis and secretion. The liver produces several components of blood plasma and provides the other tissues with vital products including nutrients and antioxidants that can keep them alive in starvation and other stress conditions. Even the discharge of potentially toxic endogenous or exogenous small molecular weight molecules occurs via synthetic processes in hepatocytes.

In this review, we focus on how the liver cell manages its glucose metabolism and NADPH pool while adapting to changes in nutrient supply, xenobiotic load, redox and antioxidant status as well as the general physiological and pathological requirements of the body. The ER is the key synthetic and secretory organelle. The multiple interactions of the hepatic ER and the liver-specific regulations of the ER homeostasis form the structural and functional basis of a crucial metabolic hub [7, 8]. ER-associated ribosomes constitute an essential component of translational homeostasis through the synthesis of secretory and cell surface proteins, as well as proteins of the secretory pathway and lysosomes in the hepatocytes. ER-glycogen interactions, which compose the hepatic glycogenoreticular system [9], are particularly important for the unique glucose metabolism of hepatocytes and are largely responsible for the distribution of glucose for different purposes [10]. ER-lysosome interactions, however, are related mainly to the regulation of autophagy [11].

The absorption, quantity and composition of the diet, in particular the presence of foreign, non-utilizable compounds, are the drivers of the short- and long-term regulation of liver function. A fundamental question is how the excess of glucose absorbed from the diet affects metabolic and redox homeostasis in hepatocytes, the liver and the body. Persistently increased dietary intake generates significant changes in liver metabolism, which are important in the development of obesity. Consequences may include the development of Metabolic Dysfunction-Associated Steatotic Liver Disease (MASLD) [12], and insulin resistance [13], and pathological conditions due to altered redox balance include Drug-induced Liver Injury (DILI) or even chemical carcinogenesis [14]. All of these are associated with impaired supply of reducing energy and altered organelle homeostasis in hepatocytes and are mediated by ER stress, oxidative stress and ferroptosis, etc., [8].

## Utilization of excess glucose absorbed from food

Glucose is a universal nutrient; however, its hydrophilic nature is a major constraint for storing large amounts, in contrast to hydrophobic fat, which can be carried without the weight of associated water. While glucose can be readily converted to fatty acids for efficient energy storage in the fed state, mobilized fatty acids cannot be converted to glucose to supply dependent cells, i.e., to maintain blood glucose level in starvation [2]. The irrevocable nature of the conversion from glucogenic to ketogenic is one of the most important problems to be solved in connection with fluctuating nutrient supply, and the responsibility lies primarily with the liver [15, 16].

**FIGURE 1:**
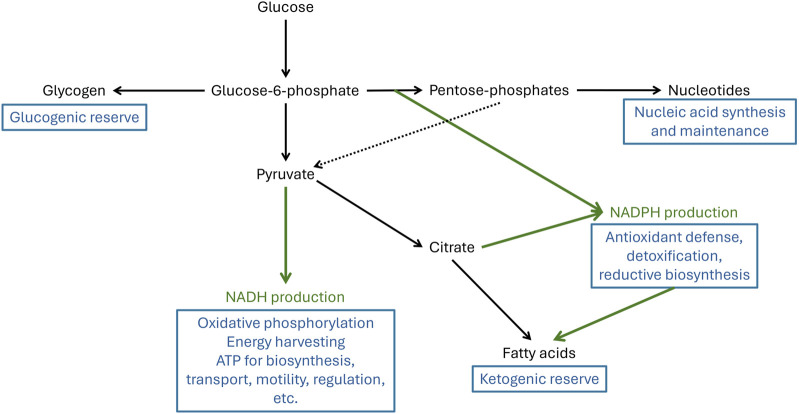
Utilization of glucose for different purposes in hepatocytes. Glucose utilization pathways branch off in three directions at the first key junction, glucose-6-phosphate. (i) Glycolysis degrades glucose to pyruvate in the cytosol. (ii) Formation of transferable glucose and its derivatives provides precursors for oligo- and polysaccharides, glycoproteins, glycolipids, glucuronides, and glycogen. (iii) Direct oxidation yields NADPH, thus supplying electrons for cholesterol and fatty acid synthesis, monooxygenation as well as antioxidant defense and detoxification. The pentose-phosphate intermediates can feed nucleotide synthesis or return to glycolysis via the pentose phosphate pathway.

**FIGURE 2:**
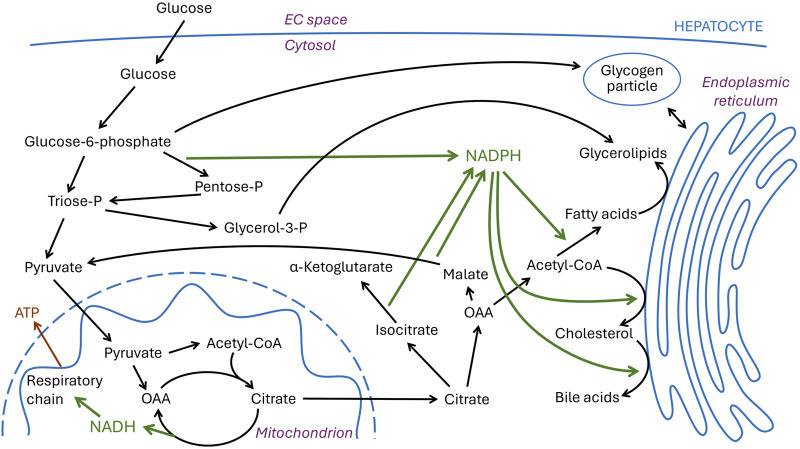
Main pathways of glucose metabolism and utilization of the reducing power in hepatocytes. Glucose replenishes glycogen stores in the fed state and enters glycolysis. Pyruvate can be (i) carboxylated to oxaloacetate (OAA) and remain glucogenic, or (ii) oxidatively decarboxylated to acetyl-CoA. The latter means an irrevocable transition to the domain of ketogenic molecules. OAA and Acetyl-CoA form citrate, which can (i) follow the track of the citrate cycle delivering electrons for oxidative phosphorylation or enter the cytosol to (ii) supply acetyl-CoA for lipid synthesis and/or (iii) provide electrons to lipid synthesis, antioxidant defense and monooxygenation through NADPH. Palmitic acid is synthesized in the cytosol, while chain elongation and desaturation, as well as the synthesis of various other lipids, are mediated by membrane-embedded enzyme systems in the endoplasmic reticulum.

Glucose can provide reducing equivalents, i.e., reduced coenzymes such as NADH or NADPH [17], for all purposes, but the liver limits its use to times of abundance. After saving as much glucose as possible by rebuilding the glycogen store, it uses electrons of the excess glucose for energy supply, i.e., ATP production in oxidative phosphorylation, detoxification by biotransformation, and maintaining antioxidant defense [18]. In addition, a large proportion of the carbon skeleton of glucose molecules, together with a large proportion of the electrons extracted from the glucose molecules, is converted into lipids, in particular fatty acids, which can be bound to glycerol and stored as fat [19]. Accordingly, hepatic glucose metabolism can be diverted at several key junctions to best adapt to the current metabolic state. One of these key junctions is the branching of pathways at glucose-6-phosphate (G-6-P) and its isomer, fructose-6-phosphate (Figure 1). The glycolytic pathway yields pyruvate, which has versatile origins and uses, thus marking another key junction (Figure 2).

Excess glucose in the fed state represents a temporary electron/hydrogen surplus in the liver. This can best be managed by channeling electrons towards the synthesis of the most reduced bio-organic molecules, fatty acids, and ultimately their most easily stored hydrophobic derivatives, triglycerides. Importantly, however, the only glucogenic part of triglycerides is the tiny glycerol, and the three fatty acid moieties will serve as a pure energy source that cannot contribute to glucose production in times of shortage [2, 19]. They can only alleviate the glucose craving of certain extrahepatic tissues by supplying ketone bodies through hepatic metabolism, hence they are referred to as ketogenic.

### Replenishing the glycogen store

Refilling glycogen stores is a high priority metabolic goal during times of plenty [10, 11, 19]. Glycogen is found in many tissues; however, the physiological function of glycogen has been studied mainly in liver and muscle, while little is known about its metabolic functions in other cells [20, 21]. Hepatocytes are one of the few cell types capable of secreting glucose, and the hepatic glycogen store serves to temporarily maintain blood glucose levels between the expiration of intestinal absorption and the onset of efficient gluconeogenesis. The utilization of hepatic glycogen is governed by a close functional interaction between glycogen granules and the ER, which has been earlier defined as the hepatic glycogenoreticular system [9]. Glycogen particles are associated with the membrane of the hepatic ER and provide the organelle with various glycogenolysis-related glucose derivatives, such as glucose-6-phosphate, UDP-glucose, UDP-glucuronic acid, gulonolactone, etc., [7, 8, 10].

### Generation of NADPH

NADPH is labelled with phosphorylation to distinguish it from NADH. Despite their essentially equivalent redox parameters, the two electron carriers have largely different metabolic roles [18]. While NADH basically transports electrons collected from various fuel molecules for energy harvesting and ATP production, the electrons of NADPH are derived from a few selected metabolic intermediates for reductive biosynthesis, maintenance of antioxidant defense and the microsomal electron transfer chains serving mostly detoxification and fatty acid desaturation (Figure 1).

The distinction between the two electron currents is also reflected in the different compartments of NADH and NADPH production. The former takes place predominantly in the mitochondrial matrix, and much of the cytosolic NADH is also shuttled there to access the electron accepting respiratory complexes on the matrix side of the inner mitochondrial membrane [17, 22]. The latter, however, is a characteristically cytosolic process, consistent with the localization of NADPH-consuming enzymes in the cytosol and on the cytosolic side of the ER membrane. Accordingly, the key metabolic junctions where the pathway forks for these two purposes of electron channeling also involve a switch of compartments. For example, citrate formed in the matrix can be utilized locally via the citrate cycle and progressively oxidized to serve ATP production or transported to the cytosol and contribute to NADPH production via the isocitrate dehydrogenase reaction (Figure 2). The latter is favored by the oversupply of citrate from glucose-derived oxaloacetate and acetyl-CoA beyond the requirements for oxidative energy harvesting in the fed state. Under such conditions, oxidation of malate molecules generated by NADH-dependent reduction of oxaloacetate in the cytosol may also switch compartment, as they can supply NADPH by oxidative decarboxylation in the cytosol rather than being transported to the mitochondrial matrix to deliver electrons to NADH and respiration (Figure 2).

Glucose is an excellent and primary source of both reducing equivalents in the liver. When the organ consumes glucose, it is because there is plenty of this nutrient, so it can provide electrons for all purposes. Since oxidative phosphorylation is adjusted to energy demand [23], antioxidant defense to oxidative challenge [24], and biotransformation to the amount of endo- and xenobiotics [25], the only electron consumer with virtually unlimited capacity is lipid, especially fatty acid synthesis. Therefore, in the fed state, the abundance of glucose virtually forces hepatocytes to enhance lipid synthesis by mass effect. This is, of course, further enhanced by simultaneous intracellular regulation and hormonal effects that promote glucose breakdown and fatty acid and triglyceride synthesis through activation of mTOR and inhibition of AMPK and PKA [26].

### Synthesis of fatty acids

Normal diet contains all the major types of nutrients, and the body faces the challenge of abundance in a fed state. It would be illogical to use dietary fats for ATP production, as fat is the fundamental means of long-term energy storage. This is why dietary triglycerides are absorbed by bypassing the portal circulation and liver into the lymphatic and systemic circulation, transported in chylomicrons and delivered directly to adipocytes for fat deposition [27]. However, the absorbed carbohydrates, mainly glucose, and amino acids collected by portal blood are also in temporary excess, and coordinating their optimal utilization and storage is an essential function of the liver.

Most of the amino acids are glucogenic, so they would come in handy for maintaining blood sugar levels during starvation [28]. However, their polymers, i.e., polypeptides, are not stored in the human body. Although general protein synthesis in the fed state is stimulated by insulin and other anabolic signaling mechanisms, and the protein balance is shifted towards degradation in starvation, and the amino acids liberated can indeed be used for gluconeogenesis in a limited period in the post-absorptive state, there is no protein in human metabolism that is destined for amino acid storage.

As mentioned above, hepatocytes build up their glycogen particles to store readily available glucose, which can be mobilized until gluconeogenic sources are accessed and glucose synthesis kicks in. When all electron-demanding pathways are saturated and glycogen stores are also full, and the liver still needs to take up glucose to avoid hyperglycemia, glucose must be converted to fatty acids and triglycerides, even if this involves a switch from glucogenic to ketogenic. A large portion of fatty acid synthesis occurs in hepatocytes from excess glucose [29]. In line with the concept that fat storage is fundamentally a conservation of energy and reducing power, the cyclic process of fatty acid synthesis involves the use of 1 molecule of ATP and 2 molecules of NADPH per pair of carbon atoms inserted in the chain. Both ATP and NADPH, in turn, are derived from the oxidative catabolism of glucose. The former is provided by the substrate level and oxidative phosphorylations in the sequence of glycolysis, PDH and the citrate cycle [19], while the latter is provided by selected dehydrogenases that oxidize glucose-6-phosphate, 6-phosphogluconate or glutamate and glucose-derived intermediates such as isocitrate or malate in the cytosol [18]. Hepatocytes thus essentially store the excess chemical energy of glucose in the most reduced and least hydrophilic form possible.

## Role of the glycogenoreticular system in different hepatic secretory functions

### Glucose secretion – maintenance of blood glucose level

The glucose and glycogen metabolism of hepatocytes is subordinated to the demands of the body. The low-affinity glucose transporter (GLUT2) allows liver cells (like other glucose-sensing cells, including the pancreatic β-cells) to detect changes in blood sugar levels [30]. As soon as a decrease in portal glucose entry is sensed, hepatic metabolism switches from glucose uptake to glucose release. The glycogen pool of the hepatocytes is designated to serve as a promptly available source of glucose production, while hormonal changes ensure that the hepatocytes have access to gluconeogenic precursors [31]. The readiness of hepatocytes to buffer fluctuations in intestinal glucose uptake is essential, and therefore replenishment of hepatic glycogen stores is a high priority whenever glucose absorption permits (Figure 2).

Dynamic mobilization of glucose from hepatic glycogen relies mainly on phosphorolysis and, to a lesser extent, autophagic lysosomal hydrolysis [32]. The process is controlled by an ER - glycogen particle - phagophore triangle [11]. In this system, glucose-6-phosphate from hepatic glycogen is channeled towards secretion via dephosphorylation in the ER. Remarkably, the altruistic action by which the hepatocyte releases glucose from the phosphate trap and allows it to leave the cell is so unusual that only a few cell types have a mechanism for liberating glucose from its phosphates. The glucose-6-phosphatase system consists of a luminal enzyme and three auxiliary membrane transporters in the ER [11, 33]. The hepatic glycogenoreticular system is in fact unique, making the hepatocyte the only cell type that can efficiently convert significant amounts of glycogen into blood glucose [10].

### Secretion of biotransformation products – detoxification

Enzymes catalyzing the steps of intermediate metabolism, i.e., carbohydrate, lipid, amino acid, and nucleotide metabolism, are generally characterized by high substrate selectivity. Consequently, certain molecules are not recognized by these scrupulous enzymes and can neither be degraded nor incorporated into cellular structures. However, these worthless endogenous or exogenous compounds, i.e., endobiotics or xenobiotics, may not be efficiently eliminated from the body and may be harmful by accumulation. The primary organ for their detoxification is the liver, and in this function, the glycogenoreticular system again gains center stage. The strategy for this task, known as biotransformation, is based on synthesis and secretion, as for many other liver functions. Biotransformation enzymes have broad substrate specificity. They usually create or liberate chemical functional groups in the original molecules (phase I) and then conjugate them with various endogenous groups (phase II). The latter usually not only diminishes the biological effect and thus attenuates toxicity but also makes the conjugate more suitable for secretion from the cell and excretion into the bile or urine (phase III). This, essentially synthetic process consumes large amounts of reducing power (NADPH) and glucuronate, both derived from glucose and glucogenic molecules [14, 34]. In addition, the main reactions of phase I and II of biotransformation take place in the membrane or lumen of the ER. Cytochrome P450 isoenzymes are integral proteins of the ER membrane facing the cytosol. They catalyze monooxygenations at the expense of O_2_ and NADPH. Remarkably, drug metabolism in the liver can be responsible for up to 30% of hepatic oxygen consumption, which also indicates a very intense NADPH demand. The quantitatively most important conjugation (phase II) reaction is glucuronidation. This process is catalyzed by UDP-glucuronosyltransferase isoenzymes (UGTs), which are also integral proteins of the ER membrane but face the lumen of the organelle [35, 36]. UGT activity requires membrane transporters for the entry of substrate UDP-glucuronate and the exit of the resulting glucuronides. Although many drug molecules and drug intermediates are exogenous substrates for UGTs, hepatic glucuronidation is always busy with the major endobiotic substrate, bilirubin.

Not only do both glucose production and glucuronidation take place in the luminal compartment of the ER, but both rely on glycogenolysis for substrate supply [34]. The hepatic glycogenoreticular system is therefore essential for both glucose secretion and drug glucuronidation [9]. This also implies that drug metabolism interferes with glucose production at several levels, for example through the demand for NADPH and glucose phosphates in hepatocytes [14]. It should be noted that some other, partly cytosolic conjugations are similarly dependent on the hepatic NADPH pool and/or glucogenic resources. For example, conjugation with glutathione requires NADPH-dependent maintenance of this antioxidant (see below), and conjugation with various amino acids diverts potential gluconeogenic resources towards detoxification.

### Replenishment of antioxidants – antioxidant defense

Although the use of oxygen as an ultimate oxidizing agent, i.e., electron acceptor in aerobic catabolism, is a very efficient way of extracting chemical energy, it also has its risks. Oxygen atoms form very stable water molecules once they have all the electrons they can take up. Partially reduced oxygen atoms, however, are highly reactive and tend to undergo non-enzymatic and therefore uncontrollable interactions to reach the fully reduced state [37]. The organism’s strategy to protect precious cellular components such as proteins, membrane lipids and nucleic acids from attack by reactive oxygen species (ROS) is to sacrifice readily oxidizable and easily recovered and therefore less valuable compounds, known as antioxidants, instead. The small antioxidant molecules usually undergo redox reactions with relatively low activation energies, so they can react with ROS with or without the involvement of enzymes. They have different redox potentials and thus the oxidized form of one can be reduced by the reduced form of the other, thus maintaining electron streams to satisfy the electron craving of harmful ROS. The source of these electron currents is NADPH, which in turn is derived from glucose metabolism, i.e., the direct oxidation of glucose and/or the dehydrogenation of certain glucogenic substances (Figure 2).

The best-known example of a redox pathway linking NADPH and H_2_O_2_ is the ascorbate-glutathione cycle, also known as the Foyer-Halliwell-Asada pathway, which is also linked to a branch containing the major lipid-soluble antioxidant alpha tocopherol, i.e., vitamin E [38, 39]. Redox cycles linking small-molecule water-soluble and lipid-soluble antioxidants play a special role in ER luminal thiol oxidation and oxidative protein folding [40–43]. Of all the tissues and organs, the liver has an outstanding capacity to synthesize glutathione and secretes this antioxidant into the blood plasma and bile [44]. Furthermore, the liver also secretes vitamin E into the sinusoidal plasma in very low density lipoproteins (VLDL).

Although ascorbate is a vitamin for humans, it is noteworthy that in species capable of producing ascorbate, the synthesis known as the hexuronic acid pathway also originates from glycogen-glucose metabolism in the liver [45–48]. Glycogenolysis has been shown to be the major source of UDP-glucuronate, and thus its intensity determines the rate of the hexuronic acid pathway [45]. In turn, the activity of glycogenolytic enzymes is regulated by the redox state of glutathione, i.e., the ratio of reduced glutathione to oxidized glutathione disulfide [44, 47]. Therefore, the metabolism of glycogen, ascorbate, and glutathione is closely related in many ways and is linked to the intracellular organelle homeostasis, particularly the glycogenoreticular system in hepatocytes [7, 9].

## Reducing power for synthesis and secretion

### Fatty acid synthesis and secretion of fat-loaded lipoproteins

Nutrient fuel molecules are in excess in the fed state, and the surplus carried by portal blood puts the burden primarily on the liver. It activates those mechanisms in liver cells that serve to store the reducing energy. The electrons in excess to those satisfying the cells' current demand for ATP, antioxidants, detoxification, etc., are directed towards synthesis as a means of storing reducing power. This implies the production of bio-organic molecules whose carbon atoms are in the most reduced state, carry the largest amount of transferable electrons in the smallest mass and space, and even lack the heavy hydrate coats, i.e., hydrophobic triglycerides. This is the energetic and electrochemical explanation for the enhancement of fatty acid synthesis, processing and esterification in the fed state, which is of course also secured by the appropriate regulatory measures in the hepatocytes.

As mentioned earlier, the price of efficient energy storage is the loss of glucogenic potential. Endogenous fatty acids produced in the fed state are both synthesized through acetyl-CoA and degraded through acetyl-CoA. However, there is no significant pathway for the conversion of acetyl-CoA to glucose in human cells. In the shortage of glucose and glucogenic intermediates, the escape route for excess acetyl-CoA molecules is their conversion to ketone bodies [49]. Energy storage in the form of fat is therefore the build-up of a ketogenic pool.

Amphipathic fatty acids are detergents that form micelles and disintegrate biological membranes and are therefore not suitable for storage. Hydrophobic triglycerides, i.e., fat molecules, on the other hand, separate from the aqueous space and form droplets or even larger drops surrounded by a monolayer of amphipathic membrane lipids [50]. Such fat droplets may appear temporarily in hepatocytes, although these cells are not specialized to host fat depots permanently. The optimal treatment of triglycerides in hepatocytes is to package them into lipoproteins assembled around apoB100 proteins in the ER membrane. The fat-loaded hepatic lipoproteins, the nascent VLDL particles, are secreted by exocytosis into the sinusoidal plasma, where they mature and, along with chylomicron particles of intestinal origin, transport their cargo of fat towards the adipose tissue for deposition [51].

Insulin, the pancreatic hormone of abundance, not only increases glucose utilization in all cells and replenishment of glycogen stores wherever relevant but also stimulates fatty acid and triglyceride synthesis as well as VLDL production and secretion in the liver. Furthermore, it favors the uptake of VLDL-derived fatty acids into adipocytes and shifts the lipogenesis-lipolysis balance toward the former. Importantly, exogenous fatty acids from the diet and endogenous fatty acids from the liver are carried in the form of triglycerides in lipoproteins. Activation of lipolysis in the adipocytes during starvation or exercise, on the other hand, yields non-esterified fatty acids, which are transported to tissues via the blood plasma as albumin-associated so-called free fatty acids (FFA) [52]. This is why plasma FFA levels are normally low in the fed state and rise steadily with prolonged starvation and/or physical activity. High insulin concentrations coincide with low FFA levels, whereas under physiological conditions, elevated plasma FFA levels occur with low insulin concentrations [53].

### Maintenance of the redox balance

Despite highly variable conditions, hepatocytes need to maintain a stable redox balance, which is best reflected by the [NAD^+^]:[NADH], [NADP^+^]:[NADPH] and [GSSG]: [GSH] ratios. Energy metabolism is fundamentally aerobic, and the activity of catabolic dehydrogenation is coupled to oxidative phosphorylation through the flow of electrons, i.e., electron yield from metabolic intermediates is matched to the electron consumption of the mitochondrial respiratory chain, so that the [NAD^+^]:[NADH] ratio is kept high [54]. This enables the reaction catalyzed by lactate dehydrogenase to proceed towards pyruvate formation, allowing the liver to detoxify lactate, the waste product of anaerobic glucose fermentation. This reaction only reverses when the [NAD^+^]:[NADH] ratio is reduced due to hepatic hypoxia or inadequately regulated metabolism of ethanol or fructose (see pathological consequences) [54]. The reaction catalyzed by glycerol-3-phosphate dehydrogenase, however, is close to equilibrium, so that in the fed state, when glycolytic intermediates are plentiful, it produces glycerol-3-phosphate for glycerolipid synthesis, whereas in the starving state it produces dihydroxyacetone-3-phosphate to channel fat-derived glycerol to gluconeogenesis.

As mentioned before, the flow of electrons carried by NADPH is separate from the electrons that drive ATP production. Hepatocytes undertake every effort to maintain a low [NADP^+^]:[NADPH] ratio, i.e., to ensure a continuous supply of NADPH for antioxidant defense and detoxification. The cytosolic NADPH provides electrons for the regeneration of GSH from oxidized GSSG, and thus actually keeps all the major antioxidants reduced and able to protect membranes and macromolecules from ROS attacks. It also serves as a substrate for CYP450 monooxygenases, which are responsible for the majority of phase I conversions in biotransformation. The cytosolic NADP-dependent metabolite dehydrogenases all use glucogenic substances, i.e., glucose-6-phosphate, 6-phosphogluconolactone, isocitrate or malate [55]. The electrons of NADH can be shunted to NADPH by nicotinamide nucleotide transhydrogenase (NNT) which is located in the inner mitochondrial membrane and uses the energy of the electrochemical proton gradient to keep NADPH in the mitochondrial matrix in a reduced state [56, 57]. In addition, glutamate dehydrogenase can also reduce NADP^+^ to NADPH in the matrix. Since the inner mitochondrial membrane is not permeable to NAD(P)H, the above reactions in the matrix can only contribute to cytosolic NADPH supply through shuttle mechanisms that also use glucogenic intermediates, i.e., isocitrate and α-ketoglutarate. Nevertheless, NNT-dependent generation of cytosolic NADPH has been shown to play an important role in maintaining a powerful antioxidant defense.

Fed state represents a flood of electrons to be utilized in the hepatocytes. Since the electron-consuming capacity for ATP production, biotransformation and antioxidant defense is demand-driven and cannot be increased indefinitely, the excess reducing power carried by NADH and NADPH is diverted by the cell towards fatty acid and triglyceride synthesis [58]. The fatty acid synthase complex uses electrons from 14 NADPH molecules to produce each palmitate molecule [59]. In addition, the process greatly reduces the number of electrons collected, as 7 acetyl-CoA molecules escape oxidation in the citrate cycle, a loss of 7 × 4 = 28 electron pairs (3 NADH and 1 FADH2 per acetyl-CoA). Not only does it reduce substrate-level phosphorylation by skipping ATP (GTP) production in the citrate cycle, but it also increases ATP demand, since 7 acetyl-CoA molecules must be carboxylated by acetyl-CoA carboxylase for incorporation into the fatty acid chain. Glycerol-3-phosphate dehydrogenase consumes one NADH for every glycerol-3-phosphate produced to provide the backbone for triglycerides. In summary, fat synthesis is very efficient at scavenging excess electrons and is essential for maintaining redox balance in the liver during absorption.

## The special status of the ER regarding synthesis, secretion and the redox state

The ER is the core of the endomembrane system and the starting point for macromolecular secretion. Polypeptide chains must present an N-terminal signal to be translated on the surface of the rough ER and at least partially enter the lumen of the organelle. However, from this point onwards, the default destination is exocytosis, resulting in secretion into the extracellular space or, in the case of membrane-associated proteins, plasma membrane localization.

As mentioned above, the ER membrane is home to the major reactions of phase I and II biotransformation, i.e., several CYP450 isoenzymes facing the cytosol and many glucuronosyltransferase isoenzymes facing the lumen [60]. Thus, in addition to the synthesis of secretory proteins, the organelle also plays a primary role in the synthesis of endobiotic and xenobiotic conjugates to be secreted. It is relevant that, in addition to the enzyme systems involved in fatty acid elongation and desaturation, enzymes synthesizing amphipathic membrane lipids and hydrophobic fatty acyl esters such as cholesteryl esters, di- and triglycerides are also localized in the ER membrane [61]. Thus, fat droplets grow between the two layers of the ER membrane, where lipid droplet organelles may develop before being isolated into the cytoplasm or, more preferentially for hepatocytes, VLDL particles may assemble and be isolated into the lumen for secretion.

While the NADP^+^-NADPH redox pair is tightly linked to the disulfide-thiol redox system of glutathione and proteins throughout the cellular compartments, and both are shifted in the direction of reduction, this is not the case in the ER. The 3D structure of proteins in the secretory pathway and proteins exposed on the cell surface or released from the cell is stabilized by intrachain and interchain disulfide bridges, which are formed in the ER lumen by oxidation of cysteinyl thiol groups as a final seal of the so-called oxidative protein folding. This requires a unique thiol-oxidizing microenvironment maintained by enzymatic thiol oxidation, which implies local uncoupling from reduced pyridine nucleotides [62]. Indeed, the absence of ER-luminal glutathione reductase, thioredoxin and thioredoxin reductase enzymes allows the peaceful coexistence of oxidized disulfides and reduced NADPH in this compartment [63]. The specific local conditions also confer a special function to ascorbate and tocopherol in the oxidative folding-related electron transfer in the ER [64].

## Pathological implications

The liver receives a highly versatile input via portal blood and has great flexibility to adjust its metabolism to stabilize certain parameters of the output. It is safe to say that liver cells are exposed to the greatest variations in their nutrient supply. The electron flow from glucose and amino acids is properly regulated by feedback at many key enzymes, so that the excess over the requirement can be channeled into fat synthesis in a well-regulated manner. However, the diet may contain components that are not supposed to be among the main sources of electrons and are accordingly oxidized by less tightly regulated enzymes. One of these is fructose, which is converted to triose phosphates by bypassing the main regulatory step in glycolysis, phosphofructokinase 1 [65]. The other is ethanol, which is oxidized to acetate by alcohol dehydrogenase and aldehyde dehydrogenase, disregarding the cell’s energy charge [66]. Consumption of fructose or ethanol (or both) results in a redox shift towards NAD(P)H and dumping of acetyl-CoA [65–67]. VLDL formation and secretion cannot keep pace with fat synthesis, leading to lipid droplet accumulation and development of fatty liver [68, 69].

Using up extra dietary glucose is one of the liver’s most important metabolic duties, and the most important way of accomplishing this is through fatty acid synthesis. Like a battery can be damaged by overcharging, adipose tissue is also harmed by excessive fat load due to overeating and physical inactivity. Excessive fat storage in adults requires adipocyte hypertrophy, as the number of fat cells hardly changes. The strain often induces inflammation in adipose tissue, with increased numbers of inflammatory macrophages and other cells of the immune system releasing inflammatory mediators that interfere with insulin signaling in adipocytes [70]. Due to the large – and growing – size of the adipose tissue, the local inflammation often becomes mild systemic inflammation. Long-term disturbances in insulin signaling and carbohydrate and lipid metabolism have a detrimental effect on the body’s overall homeostasis. This includes the possible spread of initially local insulin resistance [71, 72], which is due in part to the above-mentioned generalization of inflammatory signaling [73] and in part to lipotoxicity [74]. The latter is based on the simultaneous activation of lipogenesis and lipolysis in insulin-resistant adipocytes. The resulting acceleration of fat turnover can lead to a sustained increase in plasma FFA levels, which is harmful to a wide range of cells, including hepatocytes, muscle cells and pancreatic β-cells [75]. As explained earlier, abundant FFA accompanied by high insulin concentrations is a fundamentally unphysiological combination. The availability of FFAs and the action of insulin are inherently antagonistic. Insulin favors the utilization of glucose and amino acids, including their conversion to fatty acids. If other nutrients are available, the clearance of acyl-CoA puts a strain on cellular metabolism, which can easily lead to oxidative and ER stress and accumulation of biosynthetic lipid intermediates [76, 77], potentially enhancing inflammatory signaling and aggravating insulin resistance or even causing cell death [78–80]. There is mounting evidence for the pathogenic role of lipotoxicity in the development of obesity-related diseases, such as the metabolic syndrome, type 2 diabetes mellitus, and metabolic dysfunction associated liver diseases, such as MASLD, which is considered to be the leading chronic liver condition globally.

The liver’s central role in maintaining glucose homeostasis, particularly the storage and utilization of excess glucose, represents both an essential physiological function and a potential vulnerability. Under conditions of chronic overnutrition, regulatory mechanisms that normally accommodate fluctuations in dietary intake become disrupted, predisposing to metabolic imbalance. Consequently, hepatic efforts to meet systemic metabolic demands contribute significantly to the pathogenesis of obesity-related disorders, including non-alcoholic fatty liver disease, insulin resistance, and the broader spectrum of metabolic syndrome.
